# Autism and intellectual disability in a patient with two microdeletions in 6q16: a contiguous gene deletion syndrome?

**DOI:** 10.1186/s13039-016-0299-8

**Published:** 2016-12-03

**Authors:** Daniela Strunk, Peter Weber, Benno Röthlisberger, Isabel Filges

**Affiliations:** 1 Medical Genetics, University Hospital Basel, Schönbeinstrasse 40, CH-4031 Basel, Switzerland; 2 Division of Neuropediatrics and Developmental Pediatrics, University Children’s Hospital, Spitalstrasse 33, CH-4056 Basel, Switzerland; 3 Medical Genetics, Department of Laboratory Medicine, Cantonal Hospital Aarau, Tellstrasse, CH-5001 Aarau, Switzerland; 4 Medical Genetics, University Hospital Basel and University of Basel, Schönbeinstrasse 40, CH-4031 Basel, Switzerland

**Keywords:** Autism, Intellectual disability, 6q16 deletion, GRIK2, PRDM13

## Abstract

**Background:**

Copy number variations play a significant role in the aetiology of developmental disabilities including non-syndromic intellectual disability and autism.

**Case presentation:**

We describe a 19-year old patient with intellectual disability and autism for whom chromosomal microarray (CMA) analysis showed the unusual finding of two *de novo* microdeletions in cis position on chromosome 6q16.1q16.2 and 6q16.3. The two deletions span 10 genes, including *FBXL4, POU3F2, PRDM13, CCNC, COQ3* and *GRIK2*. We compared phenotypes of patients with similar deletions and looked at the involvement of the genes in neuronal networks in order to determine the pathogenicity of our patient’s deletions.

**Conclusions:**

We suggest that both deletions on 6q are causing his disease phenotype since they harbour several genes which are implicated in pathways of neuronal development and function. Further studies regarding the interaction between *PRDM13* and *GRIK2* specifically may be interesting.

## Background

Copy number variations (CNV) play a significant role in the aetiology of developmental disabilities including non-syndromic intellectual impairment and/or autism [1–3], accounting for about 10–20% of non-syndromic patients [1, 4, 5]. In addition, mutations in numerous genes have been linked to the aetiology of intellectual disability (ID) [1] and autism spectrum disorders (ASD). Reduced penetrance and variable expressivity of CNVs and mutations add to the complexity of interpretation of causality. Therefore, although known to be highly heritable, the aetiology of ASD is still poorly understood with no single candidate gene accounting for a majority of ASD susceptibility [6–8]. Interpretation of the clinical significance of CNVs and evaluation of their contribution to the phenotype remains challenging, although CMAs are now widely used for diagnostic purposes.

We report on a patient with global developmental delay/intellectual disability (DD/ID) and autism for whom CMA analysis revealed the unusual finding of two microdeletions on chromosome 6q. Familial segregation analysis confirmed *de novo* events and cis-configuration of both deletions. Phenotype-genotype correlations of all genes within the deletions allowed to determine a likely pathogenic contribution of both deletions. Genes within both deletions are involved in ASD pathways and likely represent a contiguous gene deletion syndrome.

## Case presentation

Our patient, which was last seen at 19 years of age, was the second of three children of healthy non- consanguineous Caucasian parents. The family history was unremarkable.

He was born at term after an uneventful pregnancy. Birth measurements were all above the 90th centiles with weight of 4750 g (>P 90), length of 55 cm (>P90) and occipitofrontal circumference (OFC) of 37 cm (>P 90). Neonatal and infancy periods were unremarkable. The boy said his first words at 12 months and walked unsupportedly at 15 months of age. At age two, macrocephaly (above the 97th centiles for OFC) and stagnation of expressive speech as well as stereotypic behaviour and missing eye contact were noted.

At 6 ½ years the boy was diagnosed with infantile autism and severe intellectual disability. The results of the autism diagnostic observation schedule (ADOS) were above the cut-off for autism. In the Vineland adaptive behaviour scale he showed a severe developmental delay (developmental quotient 30); his social behaviour and communication behaviour met the level of a 1 ½ year old, and everyday life skills levels were comparable with a 2 ½ year old child. The boy had a very short attention span, showed stereotypic behaviour, hardly no functional or symbolic playing, almost no eye contact and minimal adaptive social skills. Expressive and receptive language skills were limited to simple orders.

Further regular follow-up exams at different ages confirmed the clinical findings. Except for a mild hypotonia neurological exams were in normal range. The patient developed dog phobia. No aggressivity or autoaggressivity was noted. Access to specialized school education and therapies as well as a stimulating familial environment allowed a certain independence in these surroundings. The patient today is able to ride a bicycle and swim. He still needs help with body hygiene.

At the last clinical exam at age 19 dysmorphic signs were a mild dolichocephalus, a low set hairline on the neck, a broad face, hypertelorism (IPD > 97 P), medial sparse eyebrows, bilateral prominent anthelices, posteriorly rotated ears, a short wide nose, full lips, widely spaced teeth, prominent upper incisors, long hands (hand length >97 P), hyperextensible joints and a slight funnel chest. Measurements taken for OFC, weight and length were above the 97^th^ centile. The parents and siblings physical parameters are the following: mother 167 cm, 64 kg (BMI 22.9 kg/m^2^), father 187 cm, 98 kg (BMI 28 kg/m^2),^ sister 1 and 2 174 cm, 58 kg (BMI 19.2 kg/m^2^) and 179 cm, 65 kg (BMI 20.3 kg/m^2^), respectively.

Apart from frequent middle ear infections the patient was in good health. Extensive investigations in childhood which included brain magnetic resonance imaging (MRI), cranial computed tomography (CT), electroencephalogram and an abdominal ultrasound all proved unobtrusive. An X-ray of the hand performed at age 10 showed a slightly retarded bone age. Conventional chromosome analysis of lymphocytes (GTG-banding, 450 band level) did not show any numerical or structural anomalies. Specific testing for Fragile – X syndrome, Prader-Willi-, Angelman- and Beckwith-Wiedemann syndromes and for a 22q13 deletion did not show any abnormal results.

## Methods and results

### Chromosomal microarray

Array genomic hybridization of DNA from peripheral blood lymphocytes was performed in the patient and both his parents, using the NimbleGen WG HG18 Tiling 385 K CGHv.2.0 array. The tiling array version 2.0 contains 385,000 probes with a probe spacing of 6000 bp. Labelling and hybridization of test and reference DNA was performed according to manufacturer’s protocols.

**Fig. 1:**
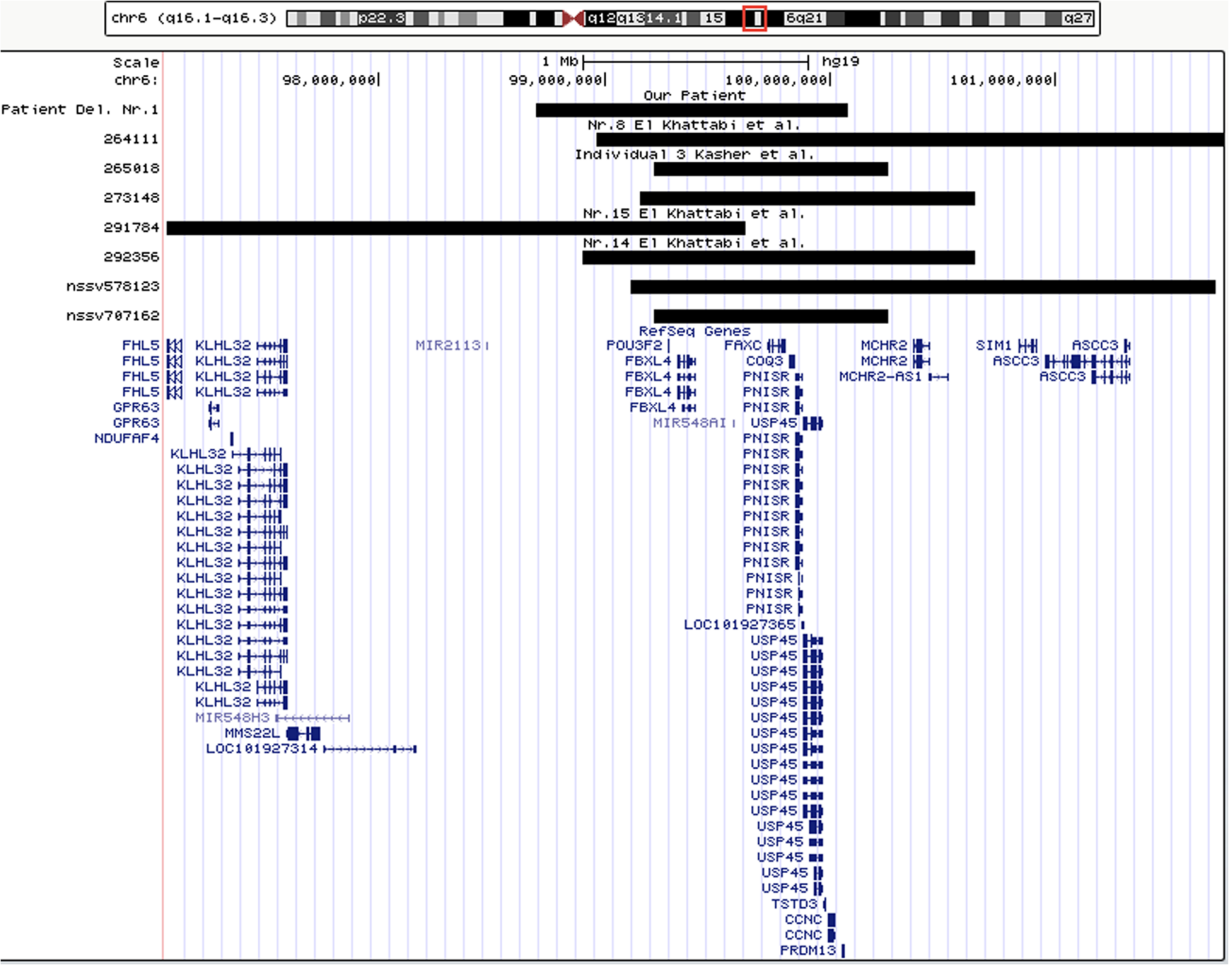
UCSC genome browser, chromosomal region 6q16.1q16.3. The black bar marked *“Patient del. Nr. 1”* shows the proximal deletion of our patient, the other black bars patients with overlapping deletions as described in Table 1. RefSeq genes are depicted in blue

The analysis identified two deletions in 6q separated by a segment of 2 Mb. The proximal deletion (Deletion Nr.1) (Fig. 1) is an interstitial deletion localized in the chromosomal region 6q16.1q16.2, spanning ~1.4 Mb (chr6: 98,693,279-100,083,279 bp, hg19 build) and encompassing nine protein coding genes of which five are listed in the Online Mendelian Inheritance in Man (OMIM) database (Table 2).

**Fig. 2:**
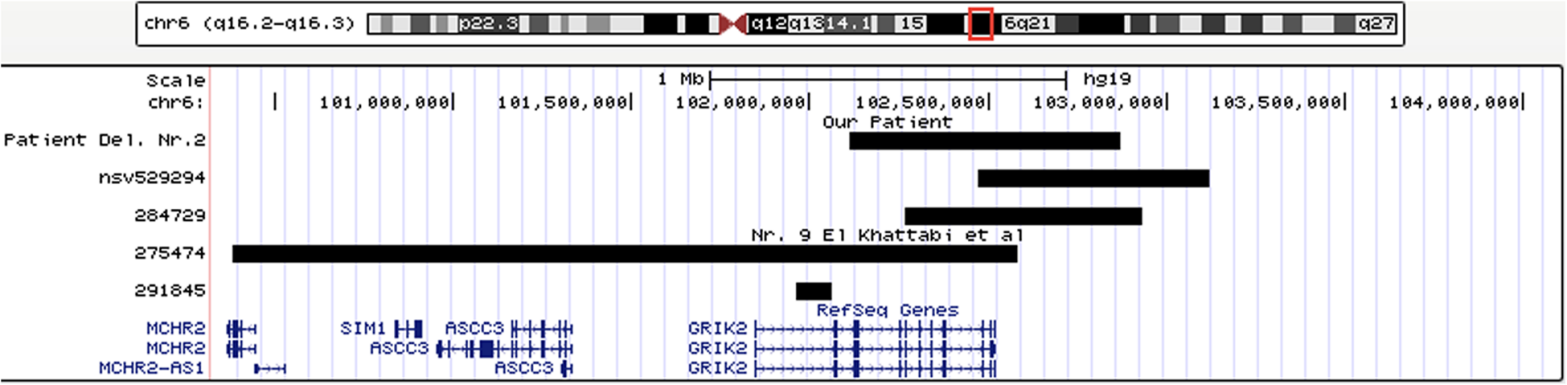
UCSC genome browser, chromosomal region 6q16.2q16.3. The black bar marked *„Patient del. Nr.2“* shows the distal deletion of our patient, other black bars patients with overlapping or flanking deletions as described in Table 1. RefSeq genes are depicted in blue

**Fig. 3:**
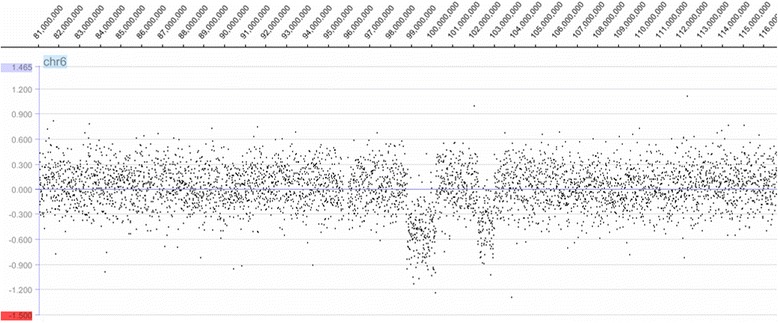
Array plot of chromosome 6q. Deletion Nr. 1 is on 6q16.1q16.2 (chr6:98693279–100083279 bp); Deletion Nr. 2 on 6q16.3 (chr6:102113307–102873307 bp). Hg version 19 (GRCh37/hg19)

The distal deletion (Deletion Nr.2), (Fig. 2) also an interstitial deletion, spans 760 kb and was assigned to the 6q16.3 chromosomal region (chr6: 102,113,307-102,873,307 bp, hg19 build). Its proximal breakpoint disrupts the *GRIK2* gene in exon 3; coding exons 4–16 are therefore deleted. Results are shown in Fig. 3.

### Fluorescence in situ hybridization

**Fig. 4:**
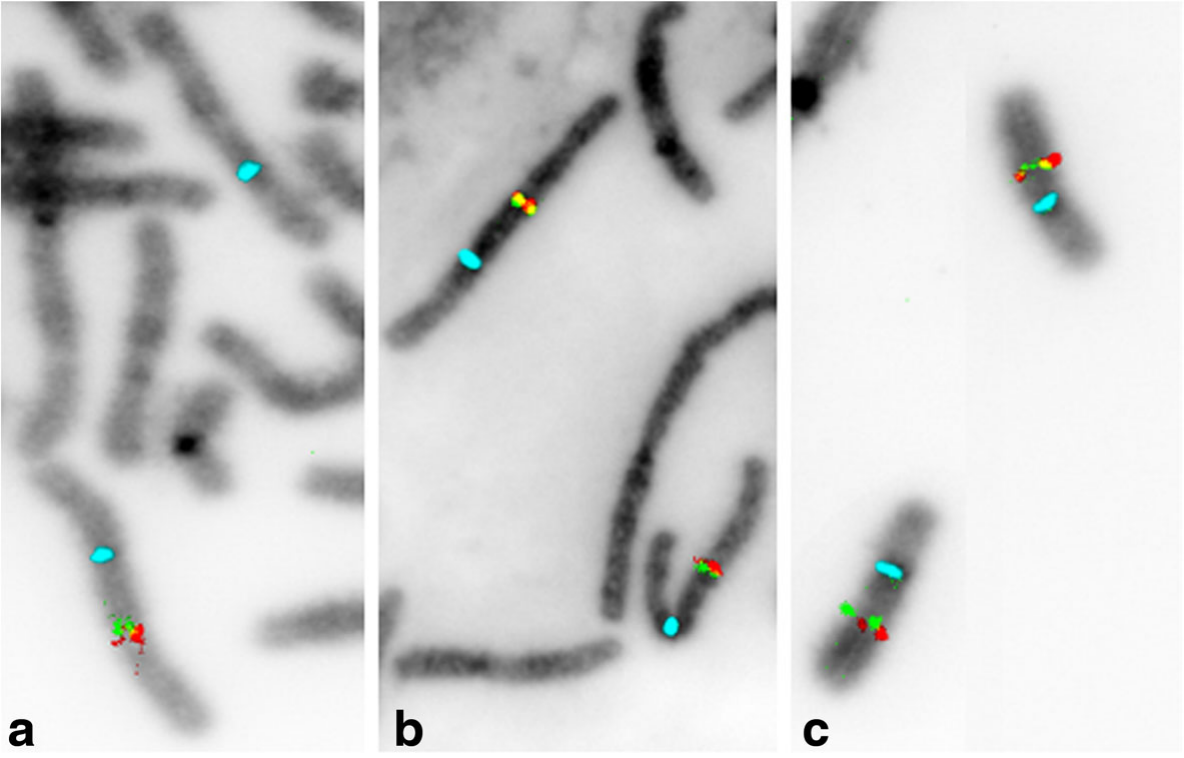
Fluorescence- in-situ-hybridization (FISH) on metaphase spreads of the patient and his parents’ chromosomes 6. We used the locus-specific Bluegnome probes RP11-758C21 for 6q16.2 (*green*), targeting deletion Nr 1, and RP11-487 F5 for 6q16.3 (*red*), targeting deletion nr.2, and the Abbott centromere specific probe cep 6 (*aqua*) for control. **a** Patient, **b** Patient’s father, **c** Patient’s mother: Both loci are present in correct orientation in the parent’s FISH analysis and proved a *de novo* origin of the deletions in the patient. Deletions in the patient are in cis position

To further investigate the structural anomaly fluorescence-in-situ-hybridization (FISH) using the BlueGnome probes RP11-758C21 for 6q16.2 (green) and RP11-487 F5 for 6q16.3 (red) was done in the patient and his parents. The deletions were confirmed to be in cis-position in the patient. FISH analysis of the parents showed both loci to be present in correct position and orientation which proved a *de novo* origin of the deletions in the patient. There was no evidence for a complex structural rearrangement in the parents. FISH results are shown in Fig. 4.

### Sanger sequencing of GRIK2

Because bi-allelic mutations in *GRIK2* have been shown to cause autosomal recessive intellectual disability [9], we sequenced all 16 exons of the *GRIK2* gene including exon-intron boundaries to confirm or rule out a compound heterozygous mutation on the second allele in *GRIK2.* No mutation was identified. Primer sequences can be obtained upon request.

### Genotype-phenotype correlations

We searched current databases, specifically Decipher [10], ClinGen [11] and DGV [12] for overlapping copy number variations in patients or healthy individuals. Results of the overlapping pathogenic deletions are shown in Table 1. We performed an extended literature and database search on all genes included in the deleted regions, their functions and related phenotypes using PubMed [13] and OMIM [14]. Results are summarized in Table 2.

**Table 1:** Overlapping deletions

Decipher ID	Variant	Interval (Mb)	Phenotypes
Overlapping deletion Nr. 1			
*Our patient*	loss: 6:98693279–100083279	1.39	posterior rotated ears, macrocephaly, autism, intellectual disability, speech impairment, hypertelorism,
*264111*	loss: 6:98966910–101858361	2.89	behavioural/psychiatric abnormality, mild intellectual disability, obesity [18]
*291784*	loss: 6:96200844–99629252	3.43	abnormality of body height, abnormality of the ear, abnormality of the nasal bridge, behavioural/psychiatric abnormality, cognitive impairment, high forehead, macrocephaly, neurological speech impairment, overgrowth [18]
*Variant Call ID* *nssv707162* *(nsv533449)*	loss: 6:99218523–100260987	1.04	abnormality of the heart global developmental delay
*dbVar ID* *nsv530906* *(Variant Call ID: nssv578123)*	loss: 6:99116405–101714826	2.59	global developmental delay
*292356*	loss: 6:98905933–100642867	1.37	development delay, learning disabilities, behavioural disorders, brachycephaly, triangular face shape, unilateral cryptorchism, strabismus [18]
*265018*	loss: 6:99218535–100260996	1	neonatal hypotonia, mild motor delay, moderate learning disability, speech delay, very severe obesity (BMI 47), hyperphagia, behavioural problems [14]
*273148*	loss: 6:99156238–100644046	1.49	severe intellectual disability, lipoma of the CNS, round face, congenital muscular torticollis, plagiocephaly, thoracolumbar scoliosis
Overlapping deletion Nr.2			
*Our patient*	loss: 6:102113307–102873307	0.7	posterior rotated ears, macrocephaly, autism, intellectual disability, speech impairment, hypertolerism,
*dbVar ID* *nsv529294*	loss: 6:102474505–103122745	0.6	global developmental delay
*275474*	loss: 6:100382250–102582366	2.2	perinatal hypotonia, developmental delay, learning disabilities, behavioural disorders, hyperphagia, obesity, synophris, hirsutism, small mouth [18]
*284729*	loss: 6:102266317–102931873	0.7	autistic behaviour
Flanking deletion Nr. 2			
*291845*	loss: 6:101962579–102060754	0.1	autism, moderate global developmental delay

Deletions spanning ≤3.5 Mb with a specified phenotype overlapping or flanking with the deletion in our patient as mentioned in the Decipher (decipher.sanger.ac.uk/browser) and ClinGen (www.clinicalgenome.org/data-sharing/) databases as well as referenced in the medical literature

**Table 2:** Involved genes

Chromosome	Gene/OMIM number	Patient phenotype	Gene function	Resource	Mendelian inheritance
Deletion Nr. 1					
6	*FBXL4/605654*	mtDNA depletion syndrome (infantile encephalomyopathic type)	Coding for a member of the F-Box family (targeting substrates for degradation of cellular regulatory proteins) Maintenance of mtDNA %HI score 10.28	[31–33]	recessive
6	*POU3F2/600494*	None described	POU domain transcription factor predominantly expressed in the CNS. Likely playing an important role in mammalian neurogenesis and neuronal migration in the neocortex, positive regulator of Schwann cell development, downstream target of *SIM1* %HI score 15.18	[15–17, 34]	Unknown
6	*PRDM13/616741*	North Carolina Macular Dystrophy (NCMD), no other neurologic phenotype described	Prdm transcription factor, neural specific direct target of Ptf1a, controlling balance of inhibitory and excitatory neurons in somatosensory circuits during neuronal development, Required to produce the right number of pax2+ neurons in the dorsal spinal cord by repressing excitatory cell fate %HI score 56.20	[25, 35, 36]	dominant for NCMD, otherwise unknown
6	*CCNC/123838*	None described	Member of the cyclin family, Protein kinase in the RNA polymerase II complex, cell cycle regulator %HI score 2.44	[30, 37]	Unknown, Protein involved in pathogenesis of tumour development and Alzheimer disease
6	*COQ3/605196*	None described	Encodes an O-Methyltransferase, involved in two steps of Ubiquinone (Coenzyme Q10) biosynthesis %HI score 40.90	[29, 38]	Unknown
Deletion Nr. 2					
6	*GRIK2/138244*	Intellectual disability; ASD; behavioural disorder, epilepsy, dystonia	Encodes for the glutamate receptor 6, excitatory neurotransmission in the brain %HI score 2.34	[5, 6, 9, 18, 22, 39]	recessive; susceptibility gene

Deletion 1 of our patient encompasses 9 genes, including *FBXL4, POU3F2, CCNC, USP45, PNISR, FAXC, TSTD3, COQ3* and *PRDM13*. Deletion 2 partially encompasses *GRIK2 (details see text)*. In this table only genes are listed with a known function described in OMIM (www.omim.org) or PubMed (http://www.ncbi.nlm.nih.gov/pubmed). Haploinsufficiency score (%HI) is mentioned as given in the Decipher database. HI score: predicted probability of exhibiting haploinsufficiency. High ranks 0–10% more likely to exhibit haploinsufficiency, low ranks 90–100% [40]

## Discussion

Chromosomal microarray is now widely used as a diagnostic tool to elucidate the aetiology of developmental delay, intellectual disability and autism spectrum disorders. The genetic heterogeneity, reduced penetrance and variable expressivity as well as suggested polygenic versus monogenic inheritance often renders the interpretation of the causal contribution of single rare CNVs to these phenotypes difficult. Here, we report on a now adult patient with global developmental delay/intellectual disability and autism for which CMA analysis has revealed the unusual finding of two interstitial *de novo* microdeletions in cis-position on chromosome 6q. Both deletions, spanning ~1.39 Mb and ~0.7 Mb respectively, separated by a genomic segment of ~2 Mb, have not been described before, but phenotype-genotype correlations with overlapping deletions mentioned in the medical literature and databases as well as haploinsufficiency of genes part of neuronal networks encompassed in both deletions, likely suggest their pathogenic contribution. Notably, Kasher et al. reported on several small heterozygous deletions in 6q16.1 presenting with variable developmental delay, intellectual disability and susceptibility to obesity. In one of their patients the deletion of 1.04 Mb includes the same nine genes as does the deletion Nr. 1 in the patient we describe [15]. The authors suggest a common critical region in their patients encompassing *POU3F2*.

*POU3F2/Brn2* belongs to a family of transcription factors that share a highly homologous POU domain and is highly expressed in the central nervous system [16], especially the human hypothalamus and hippocampus [15]. *POU3F2* upregulates proneuronal genes [16], promotes neurogenesis in the neocortex and is required for cortical neural migration [17]. Kasher et al. showed that in zebrafish, *POU3F2* functions as a downstream target of *SIM1* in the leptin – melanocortin – *SIM1* pathway and plays a role in the regulation of oxytocin expression in the hypothalamus. They suggest *POU3F2* being the likely causal mechanism affecting hypothalamic functions such as the control of food intake and social behaviour or learning, compatible with the human phenotypes in 6q16.1 deletions [15].

Previously, Bonaglia et al. [18], Griswold et al. [5], El Khattabi el al. [19] and Le Caignec et al. [20] reported on patients with mostly larger heterozygous deletions in 6q16 (in a range between 1.7 and 14 Mb) which do not allow specific phenotype-genotype correlations. However, their patients, the patients summarized in Table 1 and our patient share phenotypic findings such as hypotonia, intellectual disability, developmental delay as well as autistic features or behavioural anomalies reminiscent of ASD. Macrocephaly and tall stature and/or obesity are shared physical findings in some patients compatible with a contribution of the genomic region to growth regulation. El Khattabi et al. suggested an involvement of the *MCHR2* and *SIM1* genes in behavioural disorders and *SIM1* in obesity [19]. Both genes are not located within the deleted region of our patient. The normal BMI and feeding habits in our patient are in keeping with the finding of an incomplete obesity phenotype in the cohort of El Khattabi et al.

Of further interest, each of the two deletions in our patient span genes which have been described to play a significant role in the pathogenesis of autism [5, 6, 18, 21] and intellectual disability [22] due to their common roles in ASD associated pathways (Table 2). Particularly, the genes *GRIK2,* disrupted and partially deleted by deletion 2 in our patient, and *PRDM13,* encompassed by deletion 1, are functionally linked.

Barbon and co-workers showed experimental evidence that several different splicing variants exist, and splicing mechanisms lead to truncated subunit isoforms suggesting a complex GluR system [23]. Zhawar et al. found GluR6 receptor subunits to be differentially expressed with expression of the GluR6A variant specifically in brain [24]. Studies on mice lacking the GluK2 subunit of kainate receptors, for which *GRIK2* encodes, observed reduced social interaction and altered executive functions. The homozygous knockout of the *GRIK2* gene produces a lower capacity for pattern separation and an increased propensity for pattern completion [8]. Patients with homozygous mutations in *GRIK2* present with autosomal recessive intellectual disability [22]. In addition, studies on CNVs in ASD phenotypes suggest a contributory role of *GRIK2* haploinsufficiency in ASD [5, 18, 20, 21]. The small size of the deletion reported by Griswold et al. supports the hypothesis of *GRIK2* being a candidate gene for ASD.

**Fig. 5:**
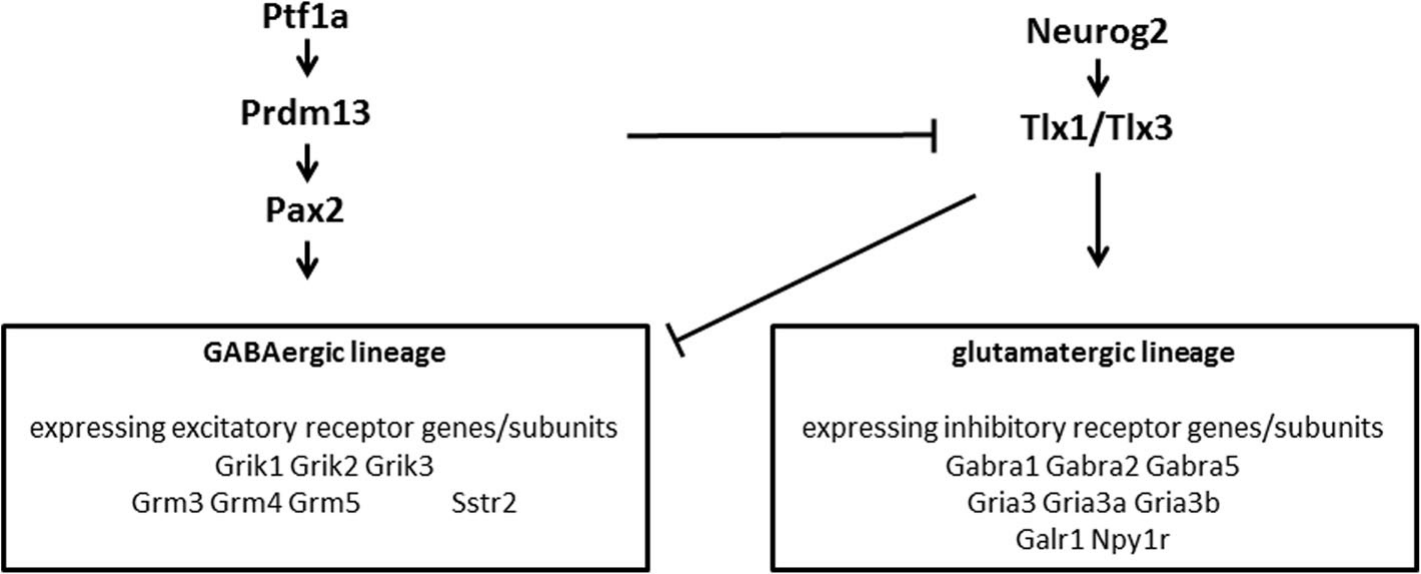
In the dorsal spinal cord PRDM13 is required to produce the correct number of pax2+/GABAergic neurons [25]. Excitatory and inhibitory neurons of the dorsal spinal cord emerge from progenitor populations within the dorsal neural tube. Depending on the transcription factors that are expressed progenitors can become neurons in either class. To redirect cell fate such a transcription factor activates lineage specific gene expression of one lineage and represses the expression of genes in the alternate lineage. The inhibitory (GABAergic) lineage of neurons for example is specified by the homeodomain (HD) transcription factor Pax2. *Pax2* and *PRDM13* are required for the specification of inhibitory cell fate in the dorsal spinal cord and are direct downstream targets of *Ptf1a*. The excitatory (glutamatergic) lineage is specified by the HD factors Tlx1/3. *PRDM13* actively represses excitatory cell fate by binding to regulatory sequences near Tlx1/Tlx3 genes, suppressing their expression. Ptf1a, a transcription activator induces factor Pax2 while suppressing the expression of Tlx1/3 which leads – through PRDM13- to an increase of inhibitory neurons at the expense of excitatory neurons. Adapted from Guo et al. [26] and Chang et al. [25]

*GRIK2* is linked to the expression of *PRDM13*, a gene involved in neuronal development. *PRDM13*, a transcription factor, is a critical component in neuronal diversity and balance of inhibitory and exhibitory neurons in the dorsal spinal cord [25]. Most interestingly, cells of the inhibitory neuronal lineage in the dorsal spinal cord express excitatory receptor genes/subunits, among them *GRIK2*, indicating an interaction between excitatory and inhibitory neurons [26] (Fig. 5).

Based on these findings haploinsufficiency of all three genes *POU3F2*, *GRIK2* and *PRDM13* indicate a contribution of both deletions to our patient’s phenotype and suggest their functional interaction as part of a contiguous gene deletion syndrome. However, the specific contribution of *PRDM13* to human disease phenotypes remains to be further elucidated.

Griswold et al. identified several novel candidate genes in molecular pathways or genetic networks implicated in autism aetiology in a large genome-wide SNP array in a European ancestry case–control data set [5]. ASD candidate genes and their functional interactions are tightly linked with general dysregulation of gene expression, disturbances of neuronal maturation and pattering and may be key in conferring susceptibility to autism spectrum conditions [27]. Multi-dimensional co-expression analysis of ASD candidate genes in the normal developing human brain suggests the heterogeneous set of ASD candidates to share transcriptional networks related to synapse formation and elimination, protein turnover, and mitochondrial function [28]. The majority of genes encompassed in deletion Nr.1 (Table 2), in particular *FBXL4*, *CCNC*, *POU3F2*, *PRDM13* and *COQ3*, can be considered part of these networks [16, 17, 25, 26, 29–31].

## Conclusions

Recent research into gene interaction and networks in autism spectrum disorders and intellectual disability suggest that phenotype-related gene products are integral to neuronal development. It often remains difficult to draw firm conclusions as to the pathogenic contribution of each single CNV or mutation of these genes. However, we suggest that the two neighbouring deletions on 6q in our patient are likely to cause his ID/ASD phenotype since they harbour several genes implicated in different linked pathways of neuronal development and function.

## Abbreviations

aCGH: Array comparative genomic hybridization
ADOS: Autism diagnostic observation schedule
ASD: Autism spectrum disorders
BMI: Body mass index
Bp: Base pairs
CMA: Chromosomal microarray analysis
CNV: Copy number variation
CoQ10: Coenzyme Q10
CT: Computer tomography
DD: Developmental delay
DGV: Database of genomic variants
DNA: Desoxyribonucleic acid
FISH: Fluorescence in-situ hybridization
GABA: Gamma-aminobutyric acid
GluK2/GRIK2: Glutamate receptor ionotropic, kainate 2
ID: Intellectual disability
IPD: Interpupillary distance
kb: Kilobases
Mb: Megabases
MRI: Magnetic resonance imaging
OFC: Occipitofrontal circumference
OMIM: Online mendelian inheritance in man
P: Percentile
SNP: Single nucleotide polymorphism

## References

[1] Filges I (2011). Deletion in Xp22.11: PTCHD1 is a candidate gene for X-linked intellectual disability with or without autism. Clin Genet.

[2] Stankiewicz P, Beaudet AL (2007). Use of array CGH in the evaluation of dysmorphology, malformations, developmental delay, and idiopathic mental retardation. Curr Opin Genet Dev.

[3] Shaw-Smith C (2004). Microarray based comparative genomic hybridisation (array-CGH) detects submicroscopic chromosomal deletions and duplications in patients with learning disability/mental retardation and dysmorphic features. J Med Genet.

[4] de Vries BBA (2005). Diagnostic genome profiling in mental retardation. Am J Hum Genet.

[5] Griswold AJ (2012). Evaluation of copy number variations reveals novel candidate genes in autism spectrum disorder-associated pathways. Hum Mol Genet.

[6] Casey JP (2012). A novel approach of homozygous haplotype sharing identifies candidate genes in autism spectrum disorder. Hum Genet.

[7] Gvozdjáková A, Kucharská J, Ostatníková D, Babinská K, Nakládal D, Crane FL (2014). Ubiquinol improves symptoms in children with autism. Oxid Med Cell Longev.

[8] Micheau J, Vimeney A, Normand E, Mulle C, Riedel G (2014). Impaired hippocampus-dependent spatial flexibility and sociability represent autism-like phenotypes in GluK2 mice. Hippocampus.

[9] Motazacker MM (2007). A defect in the ionotropic glutamate receptor 6 gene (GRIK2) is associated with autosomal recessive mental retardation. Am J Hum Genet.

[10] DECIPHER v9.3 : Mapping the clinical genome. [Online]. Available: https://decipher.sanger.ac.uk/. Accessed 17 Nov 2015.

[11] ClinGen - ClinGen | Clinical Genome Resource. [Online]. Available: https://www.clinicalgenome.org/. Accessed 17 Nov 2015.

[12] Database of Genomic Variants. [Online]. Available: http://dgv.tcag.ca/dgv/app/home?ref = GRCh37/hg19. Accessed 26 Oct 2016.

[13] Home - PubMed - NCBI. [Online]. Available: http://www.ncbi.nlm.nih.gov/pubmed. Accessed 17 Nov 2015.

[14] OMIM - Online Mendelian Inheritance in Man. [Online]. Available: http://www.omim.org/. Accessed 09 Nov 2016.

[15] Kasher PR (2016). Small 6q16.1 deletions encompassing POU3F2 cause susceptibility to obesity and variable developmental delay with intellectual disability. Am J Hum Genet.

[16] Dominguez MH, Ayoub AE, Rakic P (2013). POU-III transcription factors (Brn1, Brn2, and Oct6) influence neurogenesis, molecular identity, and migratory destination of upper-layer cells of the cerebral cortex. Cereb Cortex N Y N 1991.

[17] McEvilly RJ, de Diaz MO, Schonemann MD, Hooshmand F, Rosenfeld MG (2002). Transcriptional regulation of cortical neuron migration by POU domain factors. Science.

[18] Bonaglia MC (2008). Detailed phenotype-genotype study in five patients with chromosome 6q16 deletion: narrowing the critical region for Prader-Willi-like phenotype. Eur J Hum Genet.

[19] El Khattabi L (2015). Incomplete penetrance and phenotypic variability of 6q16 deletions including SIM1’. Eur J Hum Genet.

[20] Le Caignec C, Swillen A, Van Asche E, Fryns J-P, Vermeesch JR (2005). Interstitial 6q deletion: clinical and array CGH characterisation of a new patient. Eur J Med Genet.

[21] Jamain S (2002). Linkage and association of the glutamate receptor 6 gene with autism. Mol Psychiatry.

[22] Poot M, Eleveld MJ, van ’t Slot R, Ploos van Amstel HK, Hochstenbach R (2010). Recurrent copy number changes in mentally retarded children harbour genes involved in cellular localization and the glutamate receptor complex. Eur J Hum Genet.

[23] Barbon A, Vallini I, Barlati S (2001). Genomic organization of the human GRIK2 gene and evidence for multiple splicing variants. Gene.

[24] Zhawar VK, Kaur G, deRiel JK, Kaur GP, Kandpal RP, Athwal RS (2010). Novel spliced variants of ionotropic glutamate receptor GluR6 in normal human fibroblast and brain cells are transcribed by tissue specific promoters. Gene.

[25] Chang JC (2013). Prdm13 mediates the balance of inhibitory and excitatory neurons in somatosensory circuits. Dev Cell.

[26] Guo Z (2012). Tlx1/3 and Ptf1a control the expression of distinct sets of transmitter and peptide receptor genes in the developing dorsal spinal cord. J Neurosci.

[27] Casanova EL, Sharp JL, Chakraborty H, Sumi NS, Casanova MF (2016). Genes with high penetrance for syndromic and non-syndromic autism typically function within the nucleus and regulate gene expression. Mol Autism.

[28] Mahfouz A, Ziats MN, Rennert OM, Lelieveldt BPF, Reinders MJT (2015). Shared pathways among autism candidate genes determined by co-expression network analysis of the developing human brain transcriptome. J Mol Neurosci.

[29] Jonassen T, Clarke CF (2000). Isolation and functional expression of human COQ3, a gene encoding a methyltransferase required for ubiquinone biosynthesis. J Biol Chem.

[30] Li H (1996). Molecular cloning and chromosomal localization of the human cyclin C (CCNC) and cyclin E (CCNE) genes: deletion of the CCNC gene in human tumors. Genomics.

[31] Cenciarelli C, Chiaur DS, Guardavaccaro D, Parks W, Vidal M, Pagano M (1999). Identification of a family of human F-box proteins. Curr Biol.

[32] Bonnen PE (2013). Mutations in FBXL4 cause mitochondrial encephalopathy and a disorder of mitochondrial DNA maintenance. Am J Hum Genet.

[33] Winston JT, Koepp DM, Zhu C, Elledge SJ, Harper JW (1999). A family of mammalian F-box proteins. Curr Biol.

[34] Jaegle M (2003). The POU proteins Brn-2 and Oct-6 share important functions in Schwann cell development. Genes Dev.

[35] Hanotel J (2014). The Prdm13 histone methyltransferase encoding gene is a Ptf1a-Rbpj downstream target that suppresses glutamatergic and promotes GABAergic neuronal fate in the dorsal neural tube. Dev Biol.

[36] Hohenauer T, Moore AW (2012). The Prdm family: expanding roles in stem cells and development. Dev Camb Engl.

[37] Ueberham U, Hessel A, Arendt T (2003). Cyclin C expression is involved in the pathogenesis of Alzheimer’s disease. Neurobiol Aging.

[38] Marbois BN, Xia YR, Lusis AJ, Clarke CF (1994). Ubiquinone biosynthesis in eukaryotic cells: tissue distribution of mRNA encoding 3,4-dihydroxy-5-polyprenylbenzoate methyltransferase in the rat and mapping of the COQ3 gene to mouse chromosome 4. Arch Biochem Biophys.

[39] Córdoba M, Rodriguez S, González Morón D, Medina N, Kauffman MA (2015). Expanding the spectrum of Grik2 mutations: intellectual disability, behavioural disorder, epilepsy and dystonia. Clin Genet.

[40] Huang N, Lee I, Marcotte EM, Hurles ME (2010). Characterising and predicting haploinsufficiency in the human genome. PLoS Genet.

